# More than Cosmetic Changes: Taking Stock of Personal Care Product Safety

**DOI:** 10.1289/ehp.123-A120

**Published:** 2015-05-01

**Authors:** Rebecca Kessler

**Affiliations:** Rebecca Kessler is a science and environmental journalist based in Providence, RI.

Small glass jars full of liquid soap are neatly packed in a refrigerator-size heating chamber. Scanning the jars, Trisha Bonner reaches in and plucks one out. Unlike most of the other jars, whose contents appear thick and pearlescent, this one contains soap that has gone clear and watery. A thin layer of tiny beads, fine as sugar, dusts the bottom—exfoliating beads that have fallen out of suspension. “The scientist will be kind of sad to see this,” Bonner says.

The jar holds a prototype of one of Johnson & Johnson’s revamped Clean and Clear® facial cleansers. It’s one of hundreds of products the company is reformulating to make good on a 2011 promise to eliminate or further reduce trace amounts of several ingredients that have drawn safety concerns.^1^ Having successfully omitted substances that release small quantities of the carcinogen formaldehyde and reduced levels of the potentially carcinogenic impurity 1,4-dioxane in its baby products in 2013, the company is now working on making further changes across its baby and adult product lines by the end of this year.^1^

**Figure:**
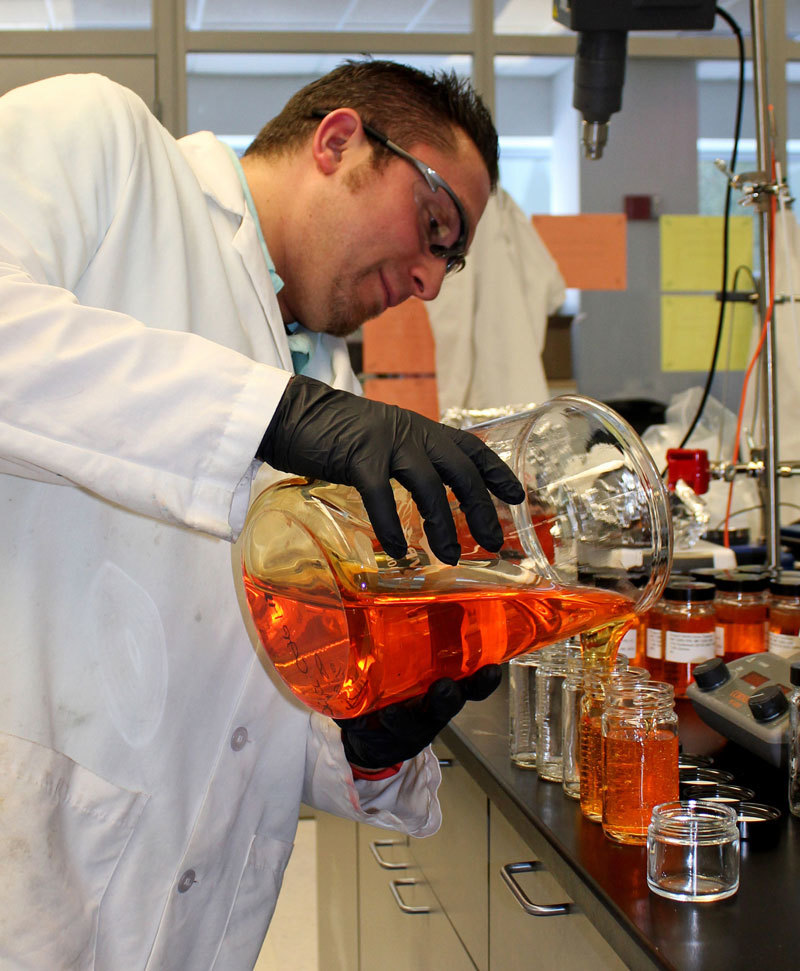
Joe Greco, principal scientist in beauty care product development for Johnson & Johnson, works on reformulating an acne gel cleanser. Johnson & Johnson is one of several companies investing considerable resources to remove specific chemicals from their products © Rebecca Kessler

The prototype in Bonner’s hand is one of many that will fail a series of tests for safety, stability, and customer satisfaction before the reformulation is complete. That’s par for the course, says Bonner, a manager of research and development at a Johnson & Johnson laboratory in rural New Jersey. Tinkering with a product’s recipe can have unintended consequences, even if only one or two ingredients are targeted for removal. And fixing one problem often sparks another.

To reformulate around 100 baby products for its 2013 deadline, the company developed 1,500 prototypes, some of which made it 18 months into the development process before failing under scrutiny, Bonner says. That initial reformulation process was particularly tricky because it involved changing the company’s most iconic products, including its baby shampoo and baby lotion, which had to retain their familiar colors, consistencies, and scents.

The present round of reformulating has been a bit easier but by no means simple, according to Cathy Salerno, vice president of research and development for the company’s North American consumer products division. For example, removing polycyclic musk fragrance ingredients, which have raised concerns as persistent and bioaccumulative endocrine disruptors,^2^ is “sort of like taking the sugar out of ice cream” because of the unparalleled warm, creamy scent they contribute, Salerno says. For certain products, rather than try to replace the musks, the company has simply jettisoned the old fragrance for a new musk-free one. In other cases, if reformulating a product becomes too troublesome, Salerno says the company might simply discontinue it.

The trail of failed prototypes notwithstanding, Johnson & Johnson is on track to meet its end-of-year deadline, Salerno says. It is also working to eliminate plastic exfoliating microbeads, which have emerged as a potentially serious water pollutant, by 2017.^1^

Under ordinary circumstances, Johnson & Johnson and other manufacturers of beauty and personal care products reformulate regularly to improve their products or because of changing ingredient availability, but this effort is much bigger. Salerno says it’s the hardest project she’s ever worked on in her 30 years at Johnson & Johnson. “We’ve never done anything on this scale before,” she says.

Johnson & Johnson’s moves have earned it praise from consumer advocacy groups as a herald of change in the cosmetics and personal care products industry.^3^ Safety concerns are transforming the industry, which in 2013 earned $41 billion in U.S. sales, according to Vera Sandarova, a spokeswoman for marketing consultancy Kline & Company. In recent years, under mounting pressure, a number of major manufacturers have begun eliminating certain controversial ingredients, and major retailers have announced plans to tailor their stock accordingly.

But even as companies such as Johnson & Johnson invest considerable resources to reformulate their products, they universally defend the questioned ingredients as perfectly safe. “It all comes down, fundamentally, to providing peace of mind to our consumers and customers. There really is no safety issue,” says Homer Swei, associate director of product stewardship at Johnson & Johnson.

Advocacy groups, on the other hand, maintain the chemicals of concern are bad news^4^ and that current regulations, which have changed little since 1938, are insufficient to protect consumers. And although the evidence for adverse health effects is still in dispute for many of these chemicals, some scientists see reason for concern.

**Figure:**
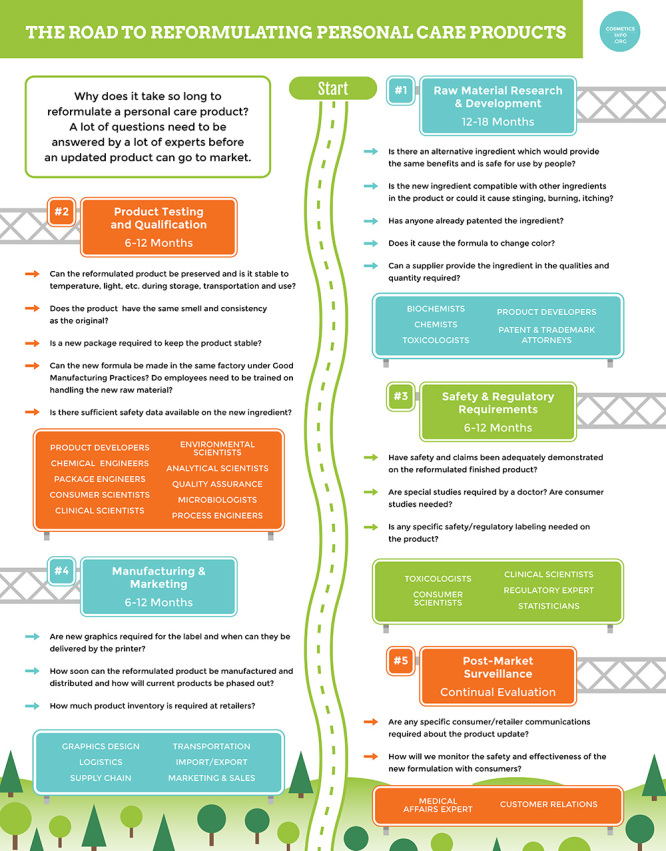
An infographic prepared by the PCPC lays out the order of events and estimated timeframe for reformulating products. © Personal Care Products Council

## Questioned Ingredients

The items most people think of as “personal care products” are generally regulated by the U.S. Food and Drug Administration (FDA) either as cosmetics or as drugs (some are considered medical devices). As defined under the Federal Food, Drug, and Cosmetic Act, cosmetics are intended to cleanse or beautify (for instance, shampoos and lipstick), while drugs are intended to diagnose, cure, mitigate, treat, or prevent disease, or to affect the structure or function of the body (for instance, sunscreens and acne creams). Some products—such as moisturizing sunscreens and anti-dandruff shampoos—may be regulated as both.^5^

The FDA can take action against companies that sell adulterated or misbranded products. However, the agency currently does not have the authority to require premarket approval of cosmetic products and ingredients, other than color additives. Manufacturers are legally responsible for substantiating the safety of their products before marketing, but the law does not require them to file any safety data or product formulations with the FDA.^6^

“To some extent, companies, in the absence of a regulator that can provide some certainty, are in a horse race to rebuild consumer trust by phasing out chemicals that have been linked to serious health problems,” says Scott Faber, vice president of government affairs at the Environmental Working Group (EWG). This Washington, DC–based advocacy group runs the popular Skin Deep online database,^7^ which grades the safety of personal care products and their ingredients.

The ingredients that receive the most attention, and are most frequently targeted for removal by manufacturers, include several known or suspected endocrine disruptors, such as diethyl phthalate (DEP), which is a common constituent of fragrance;^8^ parabens, which are widely used preservatives;^9^ and triclosan, which is an antibacterial constituent of soaps and toothpastes and a preservative in other personal care products.^10^ Also of concern are carcinogenic formaldehyde and “donor” preservatives that release it, such as quaternium-15 and DMDM hydantoin.^11^

Among the ingredients that have come under fire, parabens are causing perhaps the most concern across the industry. Parabens are among the most widely used preservatives, which are essential to maintaining the safety and stability of personal care products because they prevent microbial growth. Keeping a wide array of preservatives in use helps prevent the development of resistant microbial strains and keep people from developing sensitivities to particular preservatives, experts say.

**Figure:**
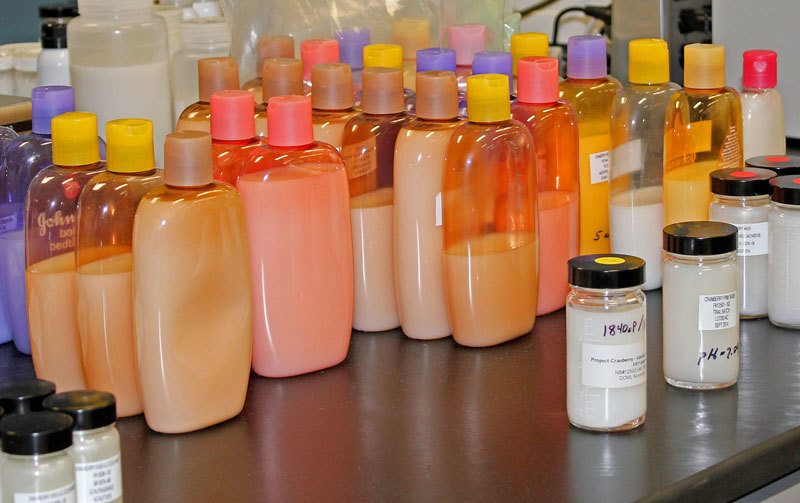
In 2013 Johnson & Johnson completed reformulation of about 100 baby products, a process that involved approximately 1,500 prototypes. Some prototypes made it 18 months into the development process before failing under scrutiny, highlighting just how complex and time-consuming the process can be. © Rebecca Kessler

“What happens is you end up looking at newer preservatives, or sometimes even older preservatives that may have been out of vogue, and really running out of options that will provide the shelf life and the stability that consumers are expecting,” says Beth Lange, chief scientist at the Washington, DC–based Personal Care Products Council (PCPC).

But substitutions aren’t simple. For instance, consumers have recently been reporting allergic reactions to a preservative called methylisothiazolinone (MI).^12^ MI is not new, but companies started relying on it more as they have turned away from parabens. People apparently became sensitized to MI through increased exposure, and some companies are now removing the chemical from some products.^13^ In addition to trying to develop new preservatives, Lange says companies are shrinking product package sizes and shortening expiry dates. But these strategies also mean stores can’t stock products as long, and consumers may end up paying more.

## Potential for Harm?

Each of the controversial ingredients has its own properties, uses, and body of literature describing safety considerations. The Cosmetic Ingredient Review (CIR), a scientific panel funded by the PCPC trade association, evaluates ingredients on the basis of animal and human safety testing data submitted by manufacturers as well as toxicity and epidemiological studies in the published literature. The panel has reviewed 3,600 cosmetic ingredients and has deemed only 11 unsafe and another 50 as having insufficient or no safety data to support use, according to Wilma Bergfeld, senior dermatologist at the Cleveland Clinic, who has chaired the panel for 25 years.^14^ Manufacturers by and large discontinued using these ingredients at CIR’s advice, and only very rarely has the FDA stepped in, says Bergfeld.

“I can honestly say in reflection that your cosmetics are safe,” Bergfeld says. “These same [ingredients] are in your food at high concentrations. There’s where you might have the greater problem.”

For a few highly scrutinized ingredients, such as DEP and parabens, the FDA has conducted its own analyses and concluded there currently is no scientific basis for taking action.^15^^,^^16^ The FDA specifically prohibits or restricts just 11 ingredients from cosmetic use.^17^ However, a cosmetic product would also be prohibited if it were harmful to consumers when used as intended, even if it did not contain an ingredient specifically prohibited by regulation.

Evidence of an adverse reaction to a cosmetic ingredient—whether from reported events or from toxicology studies *in vitro* or in animals—is not necessarily enough to support new regulations on the ingredient, says Nakissa Sadrieh, director of the FDA cosmetics division. “The dose, the route of exposure, and the biological susceptibility of individuals are some of the key factors that influence the safety of a specific ingredient in a cosmetic product,” she says. “For example, some materials are unsafe when swallowed but may be safely applied to the skin, nails, or hair.” Many of them are present in products at low levels, and some products they are found in, such as soaps, do not remain on the body long.

The agency is, however, poised to act on antibacterial agents that are used in soaps, including triclosan and a similar ingredient called triclocarban. As part of a consent decree prompted by concerns about the potential to induce antibiotic resistance and evidence of persistence in the environment and endocrine disruption in laboratory animals, in 2013 the FDA proposed a rule that requires manufacturers to provide additional safety data on the antimicrobial ingredients as well as evidence that these soaps impart a clear clinical benefit over regular soap.^18^ If they cannot provide such data, they must remove the chemicals from the soaps or relabel their products to omit the antibacterial claim.^19^ A final rule is expected by September 2016.^20^

Despite assurances from industry and the FDA that individual products are safe, the sheer number of exposures people potentially receive through personal care products raises concerns for advocacy groups and some scientists. Margie Kelly, a spokeswoman for the Campaign for Safe Cosmetics, which has been critical of both CIR and the FDA, points to an EWG survey in which people reported using an average of 9 different personal care products every day, with a quarter of women reporting using 15 or more.^21^ Some of these products, such as lipstick, are applied numerous times per day, she says. Several studies have shown a positive association between use of personal care products and increased levels of the biomarkers for DEP and certain parabens in urine and serum.^22^^,^^23^^,^^24^^,^^25^^,^^26^

**Figure:**
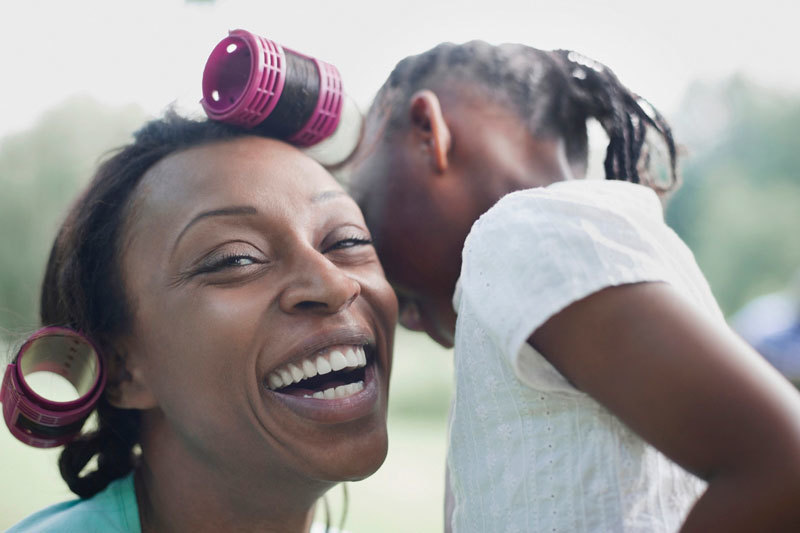
African-American women are more likely than other women to use certain types of products that may put them at greater risk for exposures to endocrine-disrupting ingredients. Major retail chains are taking steps to phase out products that use potentially harmful ingredients, but consumers who rely on smaller neighborhood beauty supply shops are unlikely to benefit from these changes. © Veer

Other studies have reported associations between exposure to DEP and earlier breast and pubic hair development in girls (risk factors for breast cancer later),^8^ neurobehavioral issues in school-age boys,^27^ and genital variations in infant boys.^28^^,^^29^ Such findings are concerning because most safety testing of cosmetics focuses on the potential for skin irritation, not higher-stakes outcomes such as cancers and reproductive problems, according to Kelly. Recent studies of endocrine disruptors challenge traditional safety-testing methodology and suggest these chemicals may induce biological responses at very low doses more often than expected.^30^

Russ Hauser, an epidemiologist at Harvard T.H. Chan School of Public Health who studies exposures to several common cosmetic ingredients, says companies are doing the right thing in removing triclosan and parabens from products, but he questions how thoroughly they have vetted the safety of the substitutions. In any case, he welcomes the attention to the broader issue of personal care product safety.

“For decades we’ve been studying what’s in the air that you breathe and the water you drink. But you wake up in the morning and you use soap, and you may use a shampoo or a conditioner, and a toothpaste, and cosmetics, and they all contain many different chemicals. And we pretty much never thought about them,” Hauser says. “I think it’s great that there’s awareness now about what’s in our everyday products. Some of the chemicals may be fine. Others may not.”

## High-Exposure Populations

There are hints that certain groups may be at greater risk of exposure than others because of the kinds of products they tend to use. Hair care products used by African-American women have drawn particular scrutiny.^31^ Hair relaxers—straightening perms—typically contain sodium hydrochloride or calcium hydrochloride, which can burn the scalp. The resulting lesions can allow the entry of other ingredients, such as the possible endocrine disruptor DEP, into the body.^32^^,^^33^ Other products once boasted that they contained estrogen or hormone-rich placenta extract to improve hair growth, although a glance at ingredient lists on some store shelves suggests manufacturers may be phasing out these ingredients.

Some studies found that African-American women were more likely than women of other ethnicities to have used hormone-containing hair care products,^34^^,^^35^ and one survey showed these products were often used on children.^36^ A 1998 case study^37^ reported that four African-American girls between ages 1 and 8 years developed either enlarged breasts or pubic hair after they began using hair products containing estrogen or placenta; laboratory analysis showed three of the products contained biologically active estriol at concentrations of 16–19 mg/g. The girls’ symptoms reversed once they stopped using the products.

African-American girls get their periods an average of six months earlier than white girls, and the causes remain under study.^38^ Because early puberty is a risk factor for breast cancer, some investigators have hypothesized that the use of hormonally active products may contribute to African-American women’s elevated risk of getting breast cancer before age 40.^39^^,^^40^ Additionally, a 2012 study found that African-American women who reported using hair relaxers were 17% more likely to be diagnosed with uterine fibroid tumors than those who did not.^41^ The study did not look at the use of specific brands or products, however, so it’s unclear what chemicals the women were exposed to. The authors also suggest that use of hair relaxers may be a proxy for use of other hormonally active products.

Tamarra James-Todd, an epidemiologist at Brigham and Women’s Hospital in Boston who has studied potential health effects of hair care products,^42^ says more research is needed to clarify connections between personal care products and poor health outcomes. Many questions will be difficult to answer without detailed product formula information, which manufacturers are not required to disclose. Yet because there is evidence that African Americans have greater exposures than other groups to endocrine disruptors (including DEP) from a variety of sources,^43^^,^^44^^,^^45^ as well as greater burdens of many hormonally mediated diseases,^46^^,^^47^^,^^48^ James-Todd says their experience warrants closer attention.

“If you only look at the subgroup of the population that has moderate levels, or even low levels of [EDC exposure], and then you try to link it to some sort of disease outcome, guess what? You might not see it,” James-Todd says. “But it might take looking at populations that have much higher levels to actually see associations.”

Nourbese Flint is program manager at Black Women for Wellness, a Los Angeles–based advocacy group that has been researching the use of hair care products in African-American homes and salons. Stylists surveyed by the group reported a variety of health problems, such as respiratory issues and skin rashes, that they attributed to the products, Flint says. Those unpublished findings, along with evidence for increased risks of neurodegenerative diseases,^49^ cancer,^50^ and respiratory problems,^51^^,^^52^^,^^53^ were underscored for salon workers across multiple ethnicities in a 2014 report by the Missoula-based advocacy group Women’s Voices for the Earth.^54^

**Figure:**
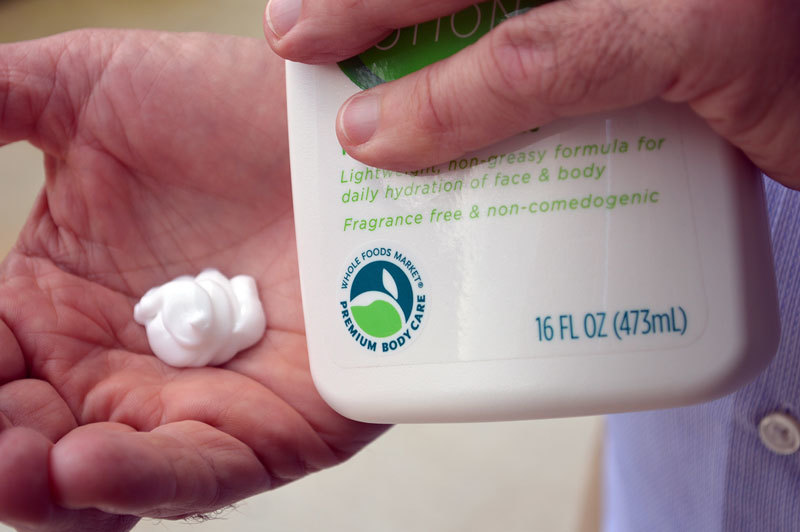
With no legal definition of advertising terms such as “natural” and “organic” as they apply to personal care products, trade groups such as the Natural Products Association and retailers such as Whole Foods are creating their own seals to indicate products meet certain standards. © Matt Ray/Brogan ∓ Partners

While Flint applauds the major retailers and manufacturers that are taking steps to reduce their use of certain ingredients, she says their actions may benefit minorities and immigrants less than general market consumers. She explains that many of the former do their shopping at neighborhood beauty supply stores that cater to specific ethnic groups, rather than at chain stores located away from inner cities. And, she says, products marketed as “safer” tend to be more expensive (although offering little guarantee that their claims are valid).

“For women who are having to make decisions about shampoo and gas and food and electricity bills … these products are just not quite accessible,” Flint says. She adds that ranking systems such as Skin Deep tend to omit products targeted at people of color, something Black Women for Wellness is working with EWG to remedy.

## Industry Takes Action

U.S. sales of “natural” personal care products grew by an estimated 7.5% in 2014, faster than the personal care product market as a whole, says Sandarova of Kline & Company. With no legal definitions for the advertising terms “natural” or “organic” in personal care products, voluntary seals have been introduced to fill the void. For example, the Natural Products Association, a trade group for manufacturers and retailers, bestows its “Natural Seal” on products whose ingredients come from “a renewable source found in nature” and have no suspected human health risks, among other criteria.^55^ The group has certified about 1,500 products and ingredients under the scheme, says Daniel Fabricant, the association’s executive director and CEO.

Ultimately, Fabricant says, developing a legal definition of the term “natural” will be essential. “There are a lot of copycats who want to use the term because they know it means something to the consumer; they know the consumer really cares about it,” he says.

In the same vein, there has also been renewed attention to ingredient labels. Some familiar chemicals that may give consumers pause, such as formaldehyde, often are not ingredients at all, but rather are released by other, more obscure ingredients, such as DMDM hydantoin. And constituents of fragrance and flavor, which include several controversial ingredients, can be considered “trade secrets” and exempt from disclosure on labels.^56^

But the PCPC’s Lange says that with tough competition among cosmetics brands, where new product launches account for roughly a fifth of annual sales, companies across the industry are responding to the pressure by eliminating controversial ingredients and opening up about their practices.

In 2007 the PCPC launched a consumer website addressing the safety of the most commonly used ingredients in cosmetics and personal care products in the United States.^57^ Johnson & Johnson maintains an extensive section on its website detailing its safety practices and ingredient policies. After Johnson & Johnson, Procter & Gamble pledged to eliminate triclosan and DEP from all its products, Avon pledged to eliminate triclosan, Colgate-Palmolive pledged to eliminate formaldehyde donors, parabens, and DEP, and at least two-dozen companies agreed to eliminate the possible human carcinogen cocamide diethanolamine.^58^^,^^59^^,^^60^^,^^61^^,^^62^

In December 2014 cosmetics giant Revlon posted a new webpage outlining its practices in regards to some two dozen ingredients.^63^ A year earlier, a negative PR campaign by advocacy groups had barraged Revlon with thousands of online petition signatures urging the company to remove specific chemicals from its products.^64^ Eventually the company engaged in a dialogue with EWG that Lucinda Treat, Revlon’s chief legal and administrative officer, describes as “very constructive.” Among other things, Revlon’s new website states that it does not use triclosan, phthalates, certain parabens, or the so-called “toxic trio” of nail polish ingredients (formaldehyde, toluene, and dibutyl phthalate); it states that it is phasing out quaternium-15 and DMDM hydantoin; and it defends its use of petrolatum and the sunscreen ingredient benzophenone-3,^63^ which has shown some evidence of endocrine disruption in aquatic environments.^65^

“Many of the positions we have in there are positions we’ve taken for some time, and there has not been a dramatic shift in our product formulas,” Treat says. What’s new, she says, is that the company publicly disclosed its practices for the first time, responding to a new expectation of transparency among consumers.

Kelly, of the Campaign for Safe Cosmetics, points out that while some manufacturers have gone public with their reformulation plans and ingredient policies, many others appear to be reformulating their products without fanfare, judging by changes her group has noted in products’ ingredient labels. “Cosmetics are absolutely safer now today than they were ten years ago,” she says. “We’re not where we need to be. But the awareness is certainly making it through to the science teams at these major manufacturers, who are clearly making adjustments to their products.”

Major retailers also are taking action. Drugstore chains CVS and Walgreens have changed their house brands or launched new ones that avoid some controversial ingredients. Whole Foods has perhaps the most comprehensive program among large retailers. Personal care products sold in Whole Foods stores must not include any of a list of 50 ingredients, and any personal care product bearing the word “organic” on the label must be certified to USDA National Organic or NSF/ANSI 305 standards. As of 2008, the store also has a stricter “Premium Body Care” standard for products that omit a list of 400 ingredients, among other criteria.^66^ In 2013 Target announced a new program for scoring personal care and other products according to such features as the safety of their ingredients, the transparency of their ingredient labels, and their environmental impact. Higher-scoring products are rewarded with incentives such as premium merchandising.^67^

The same year, Walmart announced a similar program for both Walmart and Sam’s Club stores.^68^ Suppliers of personal care and cosmetic products must submit full product formulations to a third party called The Wercs, which holds that information private. Walmart has identified a list of “priority chemicals” and a shorter list of approximately 10 “high-priority chemicals” that it says it will reduce, restrict, or eliminate in its house brands, and it encourages its suppliers to do the same.^68^ Any priority chemicals that suppliers have not eliminated must appear on product packaging starting in January 2018.^68^

However, Walmart has no plans to disclose which chemicals are on either list, according to Rob Kaplan, Walmart’s director of product sustainability, who says protecting intellectual property was a key concern in devising the policy. “Customers should know what’s in the product, and we’re giving our suppliers some time to get out of these chemicals, if they have the opportunity to,” Kaplan says. “If not, our customers should have access to that information.”

Kaplan says the change was driven by a noticeable uptick in sales of “natural” products, in conjunction with a company-wide policy of continuously improving the sustainability of its products and operations. “We’ve recognized that our customers’ needs and expectations are changing,” he says. “What they viewed as ‘performance’ and what they viewed as ‘safe’ and ‘healthy’ and ‘sustainable’ have evolved in the last several years.”

Several experts consulted for this article acknowledged “the Walmart effect” as a factor driving manufacturers to change. Kaplan says reaction to the new policy among the dozens of companies that supply Walmart’s personal care and cosmetic products has been mixed, with some companies well positioned to meet their new obligations and others sounding an anxious note about how they will comply.

Given the scale of the global personal care product supply chain, Kaplan says the industry needs to work together to advance further. To that end, in September 2014 Target and Walmart took the unusual step of jointly hosting a Beauty and Personal Care Products Sustainability Summit in Chicago.^69^ The four dozen companies that sent representatives included competing retailers and major suppliers. Kaplan says participants are now focusing on three collaborative initiatives: finding ways to increase transparency around ingredients without compromising intellectual property rights, developing criteria for evaluating sustainable chemistry in products, and developing new preservatives.

## A Possible Way Forward

Ultimately, industry and advocacy sources interviewed for this story agree that federal legislation needs revising to give the FDA more authority and resources than it currently has to regulate the cosmetics and personal care products under its purview.

“Consumers need a predictable, modern regulatory program that will ensure that chemicals in consumer products are safe. And we’re missing that,” says EWG’s Faber. “There is a lot of reformulation going on in response to tools like Skin Deep and demands by retailers like Walmart. But it makes far more sense to have the FDA act as a modern regulator than to have Walmart fill that role.”

Consumer advocates and industry groups alike have been calling for changes to the way personal care products are regulated. In recent years advocacy and industry groups have proposed competing legislation that went nowhere. And the FDA spent more than a year negotiating with the PCPC and another industry group, Independent Cosmetics Manufacturers and Distributors, over possible regulatory approaches to propose before Congress. Then, in spring 2014 the FDA ended that process with a harshly worded letter claiming the industry’s proposal “would actually reduce FDA’s current ability to take action against dangerous cosmetics,” and “could put Americans at greater risk from cosmetic-related illness and injury than they are today.”^70^

In the wake of that impasse, Senator Dianne Feinstein (D–CA) called together key groups on both sides to hammer out a legislative proposal. After more than a year in the works, and compromises on both sides, Feinstein and Senator Susan Collins (R–ME) introduced the Personal Care Products Safety Act on 20 April 2015. It has the support not only of the PCPC and several major companies but also of EWG and other advocacy groups.^71^

Among other provisions, the new bill would require the FDA to test at least five compounds per year to determine whether they are safe for use in personal care products and at what concentrations, and give the agency the power to order recalls of unsafe products. It would also require manufacturers to register with the agency and provide it with information on their products’ ingredients. With support from industry and advocacy groups, as well as Democrat and Republican cosponsors, the bill may at last pave a road forward for the entire U.S. beauty sector.
